# 

**Published:** 1888-11

**Authors:** 


NOTICE.

***All articles for publication, books for review, business communications, etc., must be sent to Editors, 1123 Spruce Street, Philadelphia.***

***Subscribers will please notice that this Journal will be sent until arrears are paid and it is ordered discontinued.***

PHILADELPHIA:

